# Doxorubicin *In Vivo* Rapidly Alters Expression and Translation of Myocardial Electron Transport Chain Genes, Leads to ATP Loss and Caspase 3 Activation

**DOI:** 10.1371/journal.pone.0012733

**Published:** 2010-09-15

**Authors:** Amy V. Pointon, Tracy M. Walker, Kate M. Phillips, Jinli Luo, Joan Riley, Shu-Dong Zhang, Joel D. Parry, Jonathan J. Lyon, Emma L. Marczylo, Timothy W. Gant

**Affiliations:** 1 Medical Research Council Toxicology Unit, Systems Toxicology Group, Leicester, United Kingdom; 2 GlaxoSmithKline PLC, Ware, United Kingdom; Buck Institute for Age Research, United States of America

## Abstract

**Background:**

Doxorubicin is one of the most effective anti-cancer drugs but its use is limited by cumulative cardiotoxicity that restricts lifetime dose. Redox damage is one of the most accepted mechanisms of toxicity, but not fully substantiated. Moreover doxorubicin is not an efficient redox cycling compound due to its low redox potential. Here we used genomic and chemical systems approaches *in vivo* to investigate the mechanisms of doxorubicin cardiotoxicity, and specifically test the hypothesis of redox cycling mediated cardiotoxicity.

**Methodology/Principal Findings:**

Mice were treated with an acute dose of either doxorubicin (DOX) (15 mg/kg) or 2,3-dimethoxy-1,4-naphthoquinone (DMNQ) (25 mg/kg). DMNQ is a more efficient redox cycling agent than DOX but unlike DOX has limited ability to inhibit gene transcription and DNA replication. This allowed specific testing of the redox hypothesis for cardiotoxicity. An acute dose was used to avoid pathophysiological effects in the genomic analysis. However similar data were obtained with a chronic model, but are not specifically presented. All data are deposited in the Gene Expression Omnibus (GEO). Pathway and biochemical analysis of cardiac global gene transcription and mRNA translation data derived at time points from 5 min after an acute exposure *in vivo* showed a pronounced effect on electron transport chain activity. This led to loss of ATP, increased AMPK expression, mitochondrial genome amplification and activation of caspase 3. No data gathered with either compound indicated general redox damage, though site specific redox damage in mitochondria cannot be entirely discounted.

**Conclusions/Significance:**

These data indicate the major mechanism of doxorubicin cardiotoxicity is via damage or inhibition of the electron transport chain and not general redox stress. There is a rapid response at transcriptional and translational level of many of the genes coding for proteins of the electron transport chain complexes. Still though ATP loss occurs with activation caspase 3 and these events probably account for the heart damage.

## Introduction

Doxorubicin (DOX), is one of the most widely used anticancer drugs for solid tumours, but its use is compromised by lifetime dose related cardiotoxicity [1]. Furthermore therapy related cardiotoxicity has become more apparent as chemotherapy becomes more successful in lengthening patient survival [2]. DOX cardiotoxicity is reproduced in several species including mice, indicating a non-species specific mechanism of toxicity [3].

Several hypotheses have been reported for the mechanism of cardiotoxicity [4]. However the most commonly described mechanism is through redox action [5]. DOX has a redox potential of −328 mV, that while not ideal for redox activity [6], does allow it to be reduced slowly by a number of redox centres in the cell [7], [8]. Additionally DOX has an affinity for cardiolipin [9], found in the inner mitochondrial membrane that can concentrate DOX in the mitochondria where it can be reduced by complex I of the electron transport chain (ETC). Overexpression of catalase decreases DOX toxicity supporting the redox hypothesis[10]. Other data though suggest the mechanism is mutifactorial, in particular involving mitochondria [11]. Inhibition of the ETC could happen through the local formation of ROS on complex I causing damage [12]. Alternatively the interaction of DOX with cardiolipin could lead to inhibition of the ETC as cardiolipin is required for normal ETC activity [13]. Furthermore, DOX can induce p53 as a result of its genotoxic properties and this in turn can lead to inhibition of the mTOR pathway that can affect mRNA translation[14]–[15]. Co-administration of redox inhibitors and ROS scavengers does not ameliorate the cardiotoxicity in the clinic also supporting the involvement of mechanisms other than, or in addition to, redox activity[16], [17].

Metabolism of DOX in the heart occurs by carbonyl reductase to form doxrubicinol [18] and overexpression of carbonyl reductase increases cardiotoxicity *in vivo* [19]. Doxorubicinol can cause the release of iron from the cytoplasmic aconitase causing the iron free aconitase to behave like iron regulatory protein (IRP) [20]. IRP causes increased levels of transferrin by mRNA stabilisation, and decreased levels of ferritin by reduced mRNA translation[21]. This may lead to increased free iron that could lead to Fenton chemistry occurring. Conversely, DOX may also inhibit IRP [20], [22] preventing binding to the iron response element (IRE). These studies indicate the effect of DOX on iron regulation in cardiomyocytes is complex. Support for an important role of altered iron homeostasis is indicated by co-administration of the iron chelating agent dexrazoxane. Dexrazoxane is partially chemoprotective for DOX cardiotoxicity and is the only approved drug for co-administration with DOX [23]. Downstream of these events loss of cardiomyocytes occurs. *In vitro* apoptosis is responsible but a role for apoptosis *in vivo* has not been conclusively demonstrated [4].

Here we used two novel approaches to add clarity to the mechanisms by which DOX initiates cardiotoxicity. First a chemically related quinone 2,3-dimethoxy-1,4-naphthoquinone (DMNQ) [24] was used as a comparator. DMNQ has an optimal redox potential of −183 mV for redox activity (thereby replicating the favored mechanistic hypothesis for DOX), and causes cardiotoxicity, but unlike DOX has only limited inhibitory effects on DNA replication and transcription. Second a global genomics approach at both transcriptional and translational levels was utilised to elucidate the biochemical pathways affected by both DOX and DMNQ. We utilised an acute dose over a short time course to avoid the complication of pathological change. We have previously published on the kinetic profiles of DMNQ[24]that reaches a C_max_ within 10 minutes in heart after i.p. administration. For doxorubicin the kinetics are different. Johansen [25] has shown in the mouse after i.p. administration of a 12 mg/kg dose of doxorubicin that maximum tissue levels are not reached until two hours. To provide some level of equivalence in this study we therefore conducted the time course of analysis for doxorubicin from 30 minutes and DMNQ from 5 minutes.

## Results

**Figure 1:**
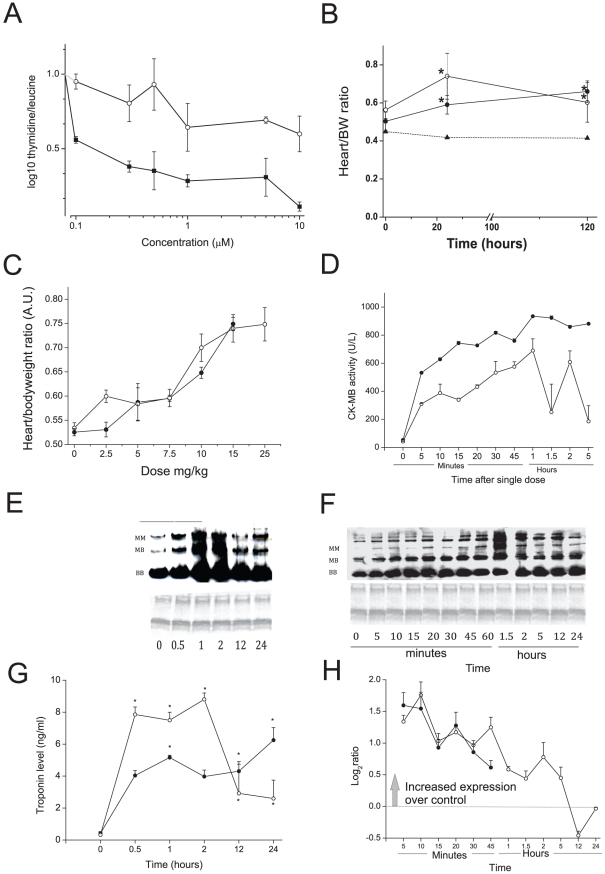
Determination of Cardiac Damage. A) Effect of DOX (filled circles) and DMNQ (open circles) on incorporation of ^3^H-thymidine/^3^H-leucine (open circles) in the mouse cardiac cell line HL-1. Incorporation was measured after 24 hr exposure to the indicated concentration. DOX significantly different from DMNQ p = 0.003 two way ANOVA. B) Heart: body weight ratio change with time after a single acute dose of DOX (15 mg/kg, filled circle) or DMNQ (25 mg/kg, open circle), or vehicle (triangle). C) Heart:body weight ratio following a dose response 120 hrs post dosing (DOX filled circles and DMNQ open circles). D) CK-MB activity determined from plasma following DOX (filled circles) or DMNQ (open circles). E and F) Native protein gels DOX (E) and DMNQ (F)probed for CK. Coomassie blue stain indicated equal protein loading. G) Troponin I activity determined from plasma following DOX (filled circles) or DMNQ (open circles) using an ELISA assay kit. H) TCap mRNA levels from the microarray data over time after acute DOX (filled circles) or DMNQ (open circles). In all graphs mean and SD are plotted. For DOX in D the SDs are very small and hidden behind the points. B,D and G: Statistical analyses performed using a one way ANOVA to time 0 with Dunnetts post-hoc t-test. * = p<0.05, except D where all points were significant.

Many hypotheses for DOX cardiotoxicity involve an element of redox cycling. In order to investigate the redox hypothesis further a biologically less complex, but redox active, quinone (DMNQ) was utilised. This allowed the redox properties of DOX to be differentiated. DMNQ has similar electro chemical characteristics to DOX but is much less able to inhibit DNA synthesis (Figure 1A). The pharmacokinetics and metabolism of DMNQ have been previously established and these data were used to design the dose and time schedules [24].

### Characterisation of cardiac damage

The model was evaluated using established biomarkers in addition to TCap mRNA, a novel biomarker identified in this study. Both DOX and DMNQ increased the heart/bodyweight (BW) ratio over a time period of 120 hr (Figure 1B). Similarly a dose response of increased heart/BW ratio was observed for 2.5 to 25 mg/kg at 120 hr after dosing (Figure 1C). A significant increase in the MB form of CK was measured from 5 min that continued to increase to 1 hr for both DOX and DMNQ (Figure 1D). Increased CK-MB was confirmed by native PAGE analysis to separate the three CK isoforms (Figure 1E and F). Plasma Troponin (TpI) was measured and a rapid increase observed after DMNQ (single 25 mg/kg) maximal at the first time point measured (30 min.) that decreased at 12 hr after dosing. The response with DOX was less acute but more prolonged (Figure 1G). Global transcriptional profiling showed the mRNA of the Titin-Cap (*TCap*) gene tracked our other markers of cardiac dysfunction very effectively. Our data indicate that expression of this gene in cardiac tissue increased with damage and is indicative of the observed pathophysiology (Figure 1H). Collectively these data indicate that rapid damage to the cardiac muscle is occurring after a single dose of either DOX or DMNQ.

### Transcriptional analysis

**Figure 2:**
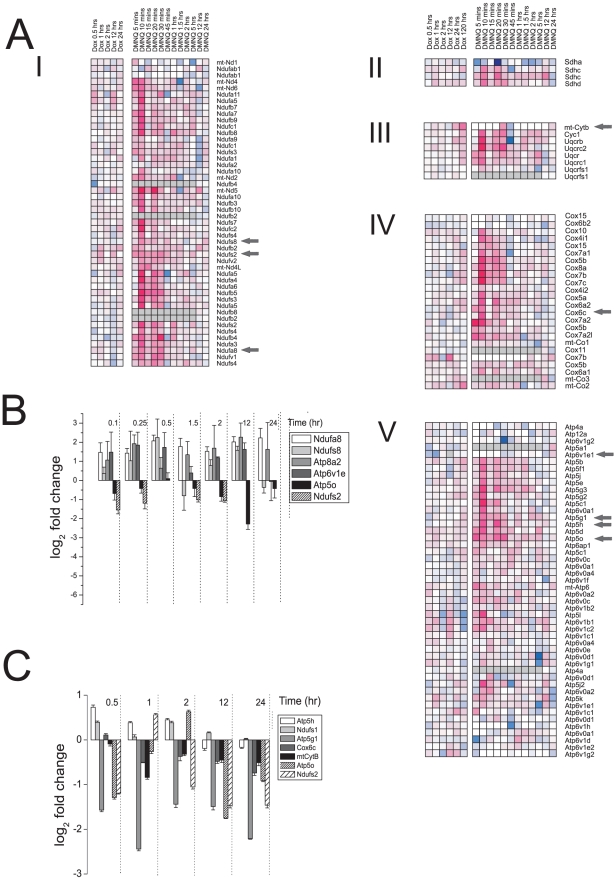
Effect of DOX and DMNQ on gene transcription in the heart *in vivo* after an acute dose over time. Microarray transcriptional analysis following acute DOX or DMNQ. A) Transcriptional gene clusters associated with each of the ETC complexes as indicated. Each cluster represents a two time courses with DOX on the left and DMNQ on the right. Genes significantly altered (p≤0.05) in expression in at least one time point were included. Magenta is increased expression and blue decreased expression with the densest colour representing a 2.5 log fold (5.6 fold) change from time matched control. Grey arrows show those genes verified by qRT-PCR in panels B and C. B) and C) qRT-PCR of ETC associated gene expression changes following DOX (B) and DMNQ (C).

Pathway analysis of transcription data indicated the functionally associated set of genes most differentially regulated in the cardiac muscle were those of the mitochondrial ETC (Figure 2A). Increased ETC gene transcription was seen, particularly for DMNQ, at the early time points after a single acute dose. After 30 min the increased transcription had resolved somewhat, corresponding with majority clearance of the DMNQ [24]. Changes in gene transcription were less marked following acute DOX that may reflect inhibition of transcription associated with DOX pharmacological action but not DMNQ. Several genes were selected for verification using qRT-PCR from each of the complexes and these are shown in Figure 2 for DOX (Figure 2B) and DMNQ (Figure 2C). Complex genes verified by qRT-PCR are indicated by grey arrows (Figure 2).

### Translational analysis

**Figure 3:**
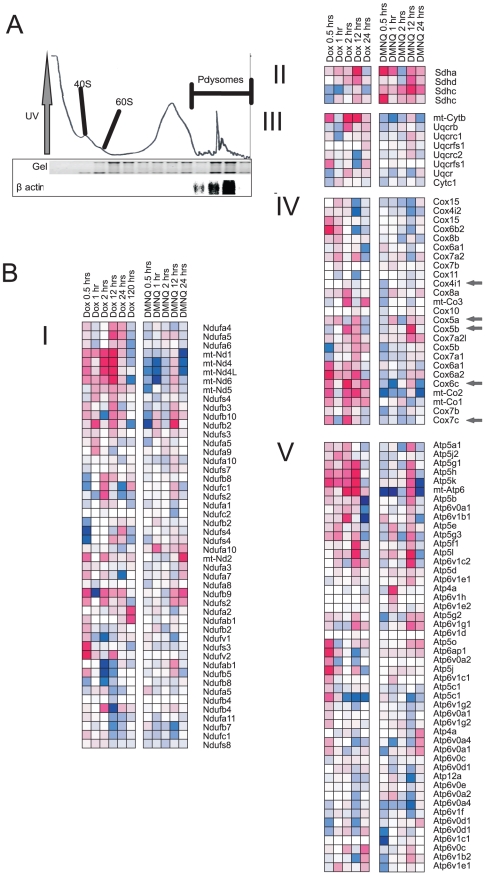
Effect of DOX and DMNQ on mRNA translation in the heart *in vivo* after an acute dose with time. Microarray translational analysis after acute DOX or DMNQ. A) UV profile of heart lysate illustrating separation of mRNA and northern blot of β-actin to show the position of the polysomes. B) Translated gene clusters associated with each of the ETC complexes as indicated. Each cluster represents two time courses with DOX on the left and DMNQ on the right. All genes that were significantly altered (P≤0.05) in expression in at least one time point were included. Magenta is increased expression and blue decreased expression the densest colour representing a 2.5 log fold change (5.6 fold) from time matched control. Grey arrows indicate those genes in complex IV regulated by NRF-1.

DOX inhibits gene transcription so we examined if regulation of mRNA translation was altered. Cardiac mRNA with attached ribosomes was fractionated on a sucrose gradient, the UV profile monitored during gradient unloading (Figure 3A) and fractions pooled according to 40S and β-actin expression (Figure 3A).The 40S mRNA level defined the monosomal (non-translated) fraction and β-actin mRNA level the polysomal (actively translated) fraction. Microarray analysis yielded similar pathways to those seen with the transcriptional analysis, in particular ETC, for both DOX and DMNQ (Figure 3B). However DOX caused a greater increase in mRNA levels through alteration of translation than DMNQ.

### Biochemical analysis of ETC complex activity

**Figure 4:**
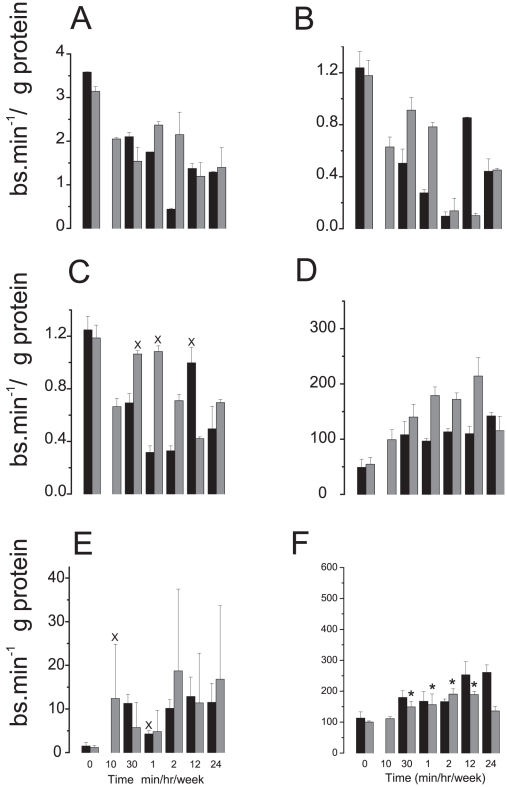
Mitochondrial complex activity in the heart *in vivo* after an acute dose of DOX or DMNQ. Single acute dose; DOX (Black) and DMNQ (Grey). Complex I to V activity is shown in Figures A to E respectively, the activity of complexes II and III was inferred by subtractions of the activity of the relevant inhibited sample from the total complex II-III assay respectively. F) Mitochondrial mass measured by citrate synthase activity. All time points compared to time 0 using a one way ANOVA with Dunnetts post hoc t-test. On all plots except F all points significantly different from time 0 except those indicated by X. For plot F * indicates significantly different from time 0 (one way ANOVA with Dunnetts post hoc t-test; p<0.05.

The activity of each ETC complex was analysed independently in cardiac muscle taken from animals over a time course following acute DOX or DMNQ. For complexes I to III activity decreased over the time course from 10 mins to 24 hr (Figure 4, A to C). In contrast activity of complexes IV and V were increased (Figure 4 D and E). To determine if any increase in mitochondrial mass had occurred the activity of citrate synthase was determined (Figure 4F). For citrate synthase a significant, increase across the time course of similar overall fold change to the increase in activity of complex IV was observed.

### Effects on cellular biochemistry

**Figure 5:**
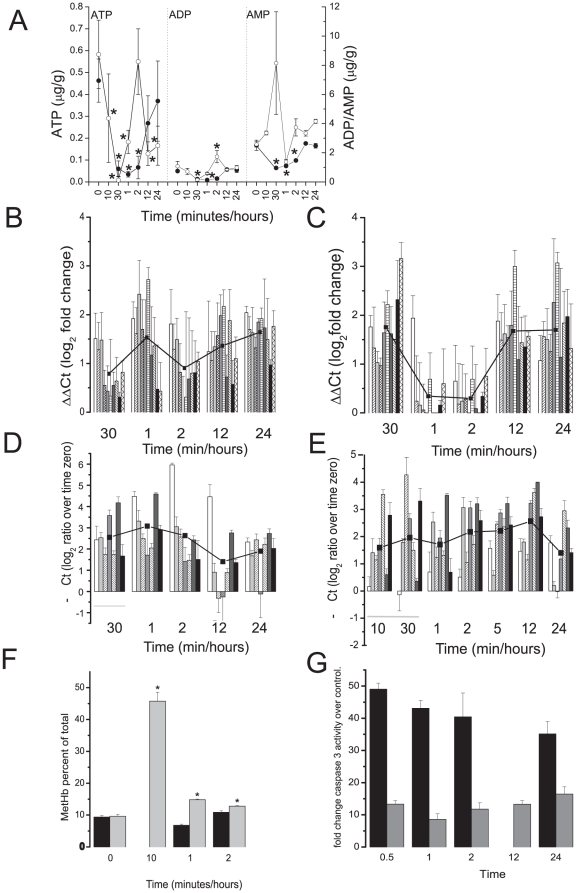
Mitochondrial associated biochemical effects following a single acute dose of DOX or DMNQ *in vivo* in cardiac tissue. A) ATP/AMP/ADP levels measured using internally controlled HPLC; DOX (filled circles) or DMNQ (open circles) * = p<0.05 one way ANOVA with Dunnetts post hoc t-test. B and C) Mitochondrial genome amplification was measured by qRT-PCR of 10 of the mitochondrial genes in cardiac tissue. From left to right the bars show the level of *ND1, CytB, Cox2, ND2, ND3, ND5, ND6, Cox3, Cox1,* and *ATP6* as a log_ 2_ fold change relative to control following treatment with DOX (B) or DMNQ (C), line is the average change at each time point. Expression AMPK complex genes; DOX (D) or DMNQ (E). Bars represent log_ 2_ fold changes relative to control and the line is the average change for all of the complexes at each time point. Left to right: *Prkaa1, Prkag3, Prkag1, Prkag2, Prkaa2, Prkab1* and *Prkab2*. F) Methemoglobin levels in the blood DOX (Black bars) or DMNQ (Grey bars) * = p<0.05 one way ANOVA with Dunnetts post hoc t-test.G) Caspase 3 activation DOX (Black bars) or DMNQ (Grey bars). Values represent fold change over control. Statistics were performed using one way ANOVA with Dunnetts post hoc t-test. For all points p<0.001.

Effects on the ETC would most likely first manifest as changes in the ATP/ADP/AMP ratios in the cardiomyocytes. After acute DOX or DMNQ there was a rapid fall in cardiomyocyte ATP (80–90%) within 10 mins post dosing. This was followed by recovery from 2 hr. DOX also induced falls in ADP and AMP levels that similarly recovered by 24 hr. DMNQ had a similar effect on ADP levels but showed an initial rise in AMP levels after dosing (Figure 5A). Overall, following a single dose of either DOX or DMNQ the AMP:ATP ratio increased whereas ATP:ADP ratio decreased. We measured mitochondrial amplification by qRT-PCR of 10 of the 13 protein coding mitochondrial genes and found an average amplification across the whole of the genome following either DOX (Figure 5B) or DMNQ (Figure 5C) treatment. The degree of mitochondrial genome amplification correlated well with the increase in citrate synthase activity (Figure 4F) suggesting both could have been due to increased mitochondrial mass. AMPK is activated by increased AMP:ATP ratio and seven genes that code for the AMPK complex (*Prkaa1, Prkag3, Prkag1, Prkag2, Prkaa2, Prkab1* and *Prkab2*) were increased over the time course from 10 to 120 min following acute DOX or DMNQ (Figure 5D and E).

Quinones are capable of causing the formation of methemoglobin by oxidizing the Fe^2+^ of hemoglobin in the process of forming the semiquinone. We measured the formation of methemoglobin after single acute doses of DOX or DMNQ. DMNQ led to methemoglobin formation at 10 mins. the point of maximum plasma concentration [24], but DOX did not (Figure 5F).

To determine if intrinsic apoptosis was occurring downstream of the mitochondrial changes we measured levels of activated caspase 3, an executioner caspase [26]. Caspase 3 was increased with acute DOX and DMNQ up to 50 fold for DOX by 30 min post dose (Figure 5G).

**Figure 6:**
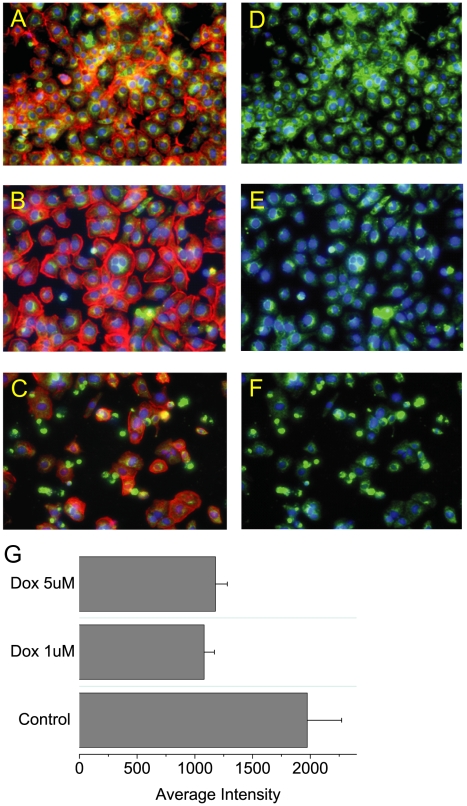
Effect of DOX on mitochondrial membrane potential and sarcomeric α-actinin expression in HL-1 cells. HL-1 cells were treated for 24 hours with 0.1% DMSO (A and D), 1 or 5 µm DOX (B–C and E–F). Mitochondrial membrane potential was assessed using mitotracker (A, C and E) and sarcomeric α-actinin by antibody (B, D and F). Green fluorescence shows mitochondrial membrane potential and red the expression of α-actinin. Where overlay of expression occurred the fluorescence appears orange.

Finally the effect of DOX on mitochondrial membrane potential and the cytoskeleton was assessed in HL-1 cells. In untreated cells there was diffuse α-actinin staining (Figure 6A) and the cells were stained green indicating a normal mitochondrial potential (Figure 6D). After exposure to DOX at 1 µM (Figure 6B and E) or 5 µM (Figure 6C and F) for 24 hours the α-actinin had condensed (Figure 6B and C) and there was a marked loss of mitochondrial potential (Figure 6E and F). At 5 µM there was substantial cell death (Figure 6C and F).

## Discussion

Many hypotheses have been advanced for DOX cardiotoxicity including redox cycling, inhibition of the ETC, iron release, activation of the mTOR pathway and induction of apoptosis (reviewed in [4]). Most studies that have led to these hypotheses have been carried out either *in vitro* or chronic dosed *in vivo* models that have limitations for genomic analysis. For the *in vitro* studies compared to those *in vivo* there are environmental differences for example oxygen tension and media composition that can result in non-specific alteration of gene expression or cell damage that may not occur *in vivo*. For chronic models there are the problems of overlying pathophysiological change that can alter the initiating insult. For these reasons we used an acute DOX model to identify events initiating the cardiomyopathy. A similar approach has been utilized previously [27]. Moreover the multiple biological and pharmacological properties of DOX can complicate the association of chemical property and biological effect. To address this we utilized DMNQ, a redox active quinone with a much weaker effect on DNA/protein synthesis compared to DOX (Figure 1A). We had already generated data on the toxicokinetics and metabolism of DMNQ [24] and therefore first demonstrated here that DMNQ causes a similar cardiotoxicity to DOX (Figure 1). We then used DMNQ to discern effects of DOX associated with inhibition of DNA replication and transcription. As DOX inhibits gene transcription directly by DNA adduct formation and probably through activation of mTOR [15] we hypothesized, and discovered here, that DOX had a limited effect on overall gene transcription compared to DMNQ. Additionally therefore we profiled mRNA translation through the use of mRNA density fractionation followed by global microarray analysis (Figure 3).

Pathway analysis for transcriptional and mRNA translational data showed for both DOX and DMNQ the most significantly altered genes were associated with oxidative phosphorylation, the ETC and actin cytoskeleton. Gene expression changes associated with these pathways became apparent from 5 min (DMNQ) or 30 min (DOX) post dosing and were prolonged (to 120 hr). Such persistent alterations in gene expression following DOX have been described previously [28]. This was of particular interest with DMNQ as the pathways remained deregulated after the compound would have been eliminated. This is concordant with metabonomics data for DMNQ in the liver that shows reduced glucose and increased lactate at 96 hr suggesting an ongoing deregulation of oxidative metabolism [24]. This would lead to cumulative effects in chronic dosing. Absent was any indication of global redox stress and so we discounted this as the mechanism of toxicity and focused on the ETC. *In vitro* it is still quite possible that redox activity is a mechanism of toxicity where oxygen tensions are higher than *in vivo* [29], [30].

**Figure 7:**
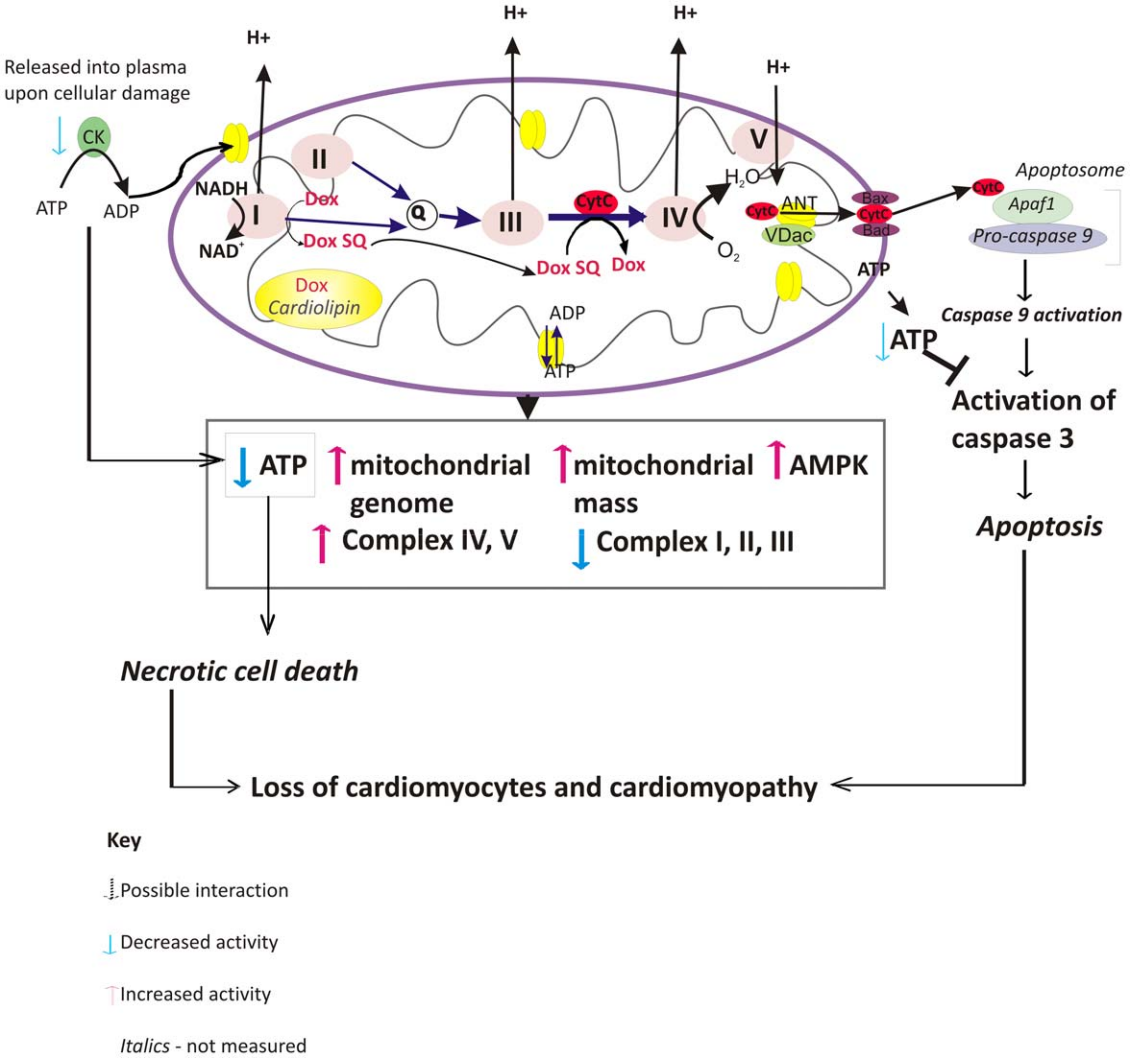
Schematic of the proposed mechanism of DOX toxicity resulting from inhibition of ATP production from the ETC. The figure shows the hypothesis of electron shunting from Complex I by doxorubicin leading to a loss of ATP and activation of caspase 3.

Biochemical analysis of each ETC complex showed that complexes I to III were reduced in activity (Figure 4) while those of complexes IV and V were increased (Figure 4 D and E) in accordance with previous analyses [12]. There was an approximate two fold increase in mitochondrial mass measured by citrate synthase activity (Figure 4F) that could account for the increased activity of complexes IV and V, but not for decreased activity of the former complexes. A possible explanation is that DOX or DMNQ was taking electrons from complex I and transporting them not to oxygen but to cytochrome c thereby short-circuiting the electron transport chain[31](Figure 7). DOX is extensively bound to cardiolipin and a pool of cytochrome c is also bound to cardiolipin in the membrane. The cytochrome c/cardiolipin complex has a greater negative redox potential than unbound cytochrome c that would diminish its ability to oxidise the semiquinone, but still its close proximity could still favor the reaction [32]. We cannot however completely discount the possibility of a local redox reaction on complex I or III. In either scenario inhibition of the ETC would reduce the ATP levels, as observed (Figure 5A). Decreased ATP leads to an activation of AMPK; we observed increased expression of the genes of the AMPK complex (Figures 5E and F) [33]. AMPK activity is also increased in response to decreased ATP to protect against apoptosis, explaining the decrease in caspase 3 activity through the time course (Figure 5G) [34]. Therefore the increased AMPK expression we observed could be an indication that the cardiomyocytes were trying to avoid apoptosis. Furthermore, reduced ATP could lead to mitochondrial damage and increased mitochondrial genome copy number or mitochondrial mass. The increase in mitochondrial genome copy number (Figure 5, C and D) appears to indicate the former but this can be partially accounted for by increased mitochondrial replication suggesting the overall response is a combination of both increased replication and genome amplification (Figure 4F). Formation of methemoglobin induced by DMNQ, particularly at the 10 min time point, indicated that DMNQ might be causing acute cardiac injury by hypoxia. Methemoglobin probably explains why PCA analysis of the whole transcriptome data plotted the 10 min. time point separated from the other time points (Supplemental Figure S1).

Gene expression changes regulated by the hypoxia inducible factor (HIF-1α) and nuclear respiratory factor (NRF-1) may indicate if hypoxia occurred with DMNQ as a result of methemoglobin formation. Genes with HIF-1α binding sites were not altered in expression but several of those regulated by NRF-1 were. NRF-1 is an important transcription factor controlling transcription of complexes of the ETC [35]. NRF-1 null mice do not survive more than 3.5 to 6.5 days post coitum [36]. Both DOX and DMNQ activated NRF-1 (Supplemental Figure S2). This increase in NRF-1 correlated with increased expression of genes with a binding site for this transcription factor (Supplemental Figure S3). Many of the Cox genes that form part of Complex IV have a binding site for NRF-1 and were increased in expression particularly with DMNQ. The activation of the NRF-1 genes with DOX and DMNQ, and not those regulated by HIF-1α, suggests a specific response of factors of the ETC and not a general response to methemoglobin formation.

Our data, and that previously published, leads us to the following hypothesis for DOX cardiotoxicity that is summarized in Figure 7. While the redox potential of DOX (−328 mv) is not ideal for redox cycling because the redox potential is too low to allow for efficient reduction by NADH:ubiquinone reductase[6], [7] there is clear electron spin resonance evidence that DOX is reduced by complex I forming the semiquinone in isolated mitochondrial preparations [37] and it seems entirely feasible that this also occurs *in vivo*. *In vitro* there is also clear evidence that the superoxide radial is sub sequentially formed completing the redox cycle [38]. *In vivo* the completion of the redox cycle may not occur. The use of cellular and subcellular preparations i*n vitro* does not necessarily mimic the conditions *in vivo* particularly in respect of oxygen tension. Most cells are cultured in conditions of 5% CO_2_ and air and will result in an oxygen partial pressure in the cells of approximately 150 mmHg [29]. In contrast within heart mitochondria the oxygen partial pressure is much lower. Data from Mik et al [39] indicates a linear relationship between oxygen tension and atmospheric concentration in rats with a partial pressure of about 60 mmHg at 20% O_2_ and a mitochondrial oxygen tension in the heart of 35 mmHg; considerably lower than the *in vitro* conditions. The overall effect of reduced oxygen tension on the redox reactions *in vivo* will be to decrease the rate of superoxide generation. Therefore while redox reactions may occur *in vivo* the amount of superoxide formed will likely be considerably less than is likely to be formed *in vitro.* Combined with effective cellular systems *in vivo* to deal with superoxide and its derivatives, and the rather non specific cellular interactions by which superoxide causes cell death, redox cycling *in vivo* does not appear totally convincing as a mechanism for DOX induced cardiotoxicity. This hypothesis is supported by clinical trials that have failed to show a benefit of antioxidants suggesting that formation of active oxygen species is not the predominant route of toxicity [16], [17]. In this study using genomics we failed to find any evidence of redox stress at either the level of transcription or mRNA translation.

While a redox signature was not seen in the genomic analysis signatures of some common gene expression pathways were quickly activated, prominent amongst them was the ETC pathway. This is a pathway commonly found induced during genomic systems analysis, and often a non-specific indicator of cellular stress. It's very rapid activation here though caused us to investigate further whether this might be a cause rather than consequence of the toxicity. Further support for investigating this pathway was gained through the use of DMNQ. As with DOX, for DMNQ *in vivo* no evidence for redox cycling was observed from the genomic analysis, but similarly to DOX a rapid induction of genes of the ETC occurred. For both DOX and DMNQ biochemical analysis indicated a loss of activity in complex I (Figure 4) commensurate with a main compound interaction with this complex, and additionally a slight increase in Complex IV activity similar to that observed previously in DOX treated rats [40]. Previous published data of rotenone inhibition of reversed ETC activity with DOX in sub mitochondrial particles showed that DOX is reduced on complex I proximal to the site of rotenone inhibition [37] supporting this hypothesis. It is interesting to speculate that while redox reactions may occur more readily *in vitro* due to the higher oxygen tension inhibition of the ETC is likely to be less effective *in vitro* as a mechanism of toxicity. This is due to cells *in vitro* generally being cultured in a high glucose concentration and utilizing glycolysis for the generation of ATP [41]. Thus it may be that while DOX could cause toxicity *in vitro* through redox reactions *in vivo* inhibition of the ETC is probably more important.

If the oxygen tension *in vivo* makes redox reactions less likely than *in vitro* there is still the question of what happens to the semiquinone once produced from reduction of the quinone by complex I. Previous studies have shown that menadione and DOX, once one electron reduced by complex I, can transfer the electron further down the ETC possibly to cytochrome c in a process known as electron shunting [31], [42], [43]. Our own work with DMNQ has shown that DMNQ is able to stimulate oxygen consumption in rotenone inhibited sub mitochondrial particles, and that this effect is cyanide sensitive indicating a similar ability (data not shown). We suggest that this mechanism is more likely to occur *in vivo* rather than production of superoxide.

Whether redox cycling or electron shunting occurs the net result will be the same, a loss of ATP (Figure 5) as there is a much greater dependency on oxidative phosphorylation to generate ATP *in vivo* than there is *in vitro*. We observed this through in the mitochondrial gene amplification, probably indicating mitochondrial fission and activation of the AMP sensitive complex AMPK which has many cellular functions associated with restoring the ATP balance in the cell [33], [44]. Ultimately this process can lead to a loss of mitochondrial membrane potential (Figure 6) and release of cytochrome c with activation of caspase 3 (Figure 5) and apoptosis (Figure 7). There is broad agreement in the literature that the loss of cardiomyocytes following DOX administration occurs by apoptosis [45], and that the subsequent stress on the remaining cells could lead to cardiomyopathy at prolonged periods of time after the DOX exposure. This has recently been elegantly shown in juvenile mice exposed to DOX that were susceptible to stress induced myocardial injury as adults [46].

If this hypothesis is correct then the most important question is, can the chemical structure of DOX be altered to give fewer detrimental cardiac effects? One possible method would be to co-administer DOX with an AMPK activator to try and maintain ATP. Another possible way forward would be to add groups that can reduce the DOX redox potential without affecting the ability of the molecule to inhibit topoisomerase II, or adduct DNA. Mitoxanthrone represents one such analog that attempts to achieve this. It has a more negative redox potential than DOX (−450 mV) and has no cardiotoxicity as a result. Unfortunately its pharmacological activity is not as favorable as that for DOX. The cannaboid derivative HU-331 has also been proposed to be more pharmacologically potent than DOX and its structure suggests a low redox potential [47]. While not ideal as chemotherapeutic agents these molecules indicate the route forward to a better DOX and support the mechanism of cardiotoxicity proposed from the data in this study.

## Materials and Methods

### Synthesis of DMNQ and animal treatment

DMNQ was synthesised according to the method of Gant et al. (1988) [48]. Dosing solutions were prepared and animal treatment performed as described in Parry et al. (2009) [24], with the exception of DOX, which was dissolved in 0.9% w/v saline. All procedures are licensed under UK Home Office project licence 80/2048. For dosing DOX was used at 15 mg/kg and DMNQ at 25 mg/kg.

### De novo protein and DNA synthesis

De novo protein and DNA synthesis was assessed in HL-1 cells by measuring the incorporation of L-[4, 5-^3^H]-leucine or [6-^3^H] – thymidine, respectively. HL-1 cells were obtained from Dr. William Claycomb (LSU Health Sciences, New Orleans, USA) and cultured according to Claycomb et al. 1998 [49]. Cells were seeded into a 96 well plate (5×10^4^/well) after treatment (0.1–10 µM DOX or DMNQ dissolved in 0.1% DMSO), media was removed and replaced with complete media containing labelled leucine or thymidine to a final concentration of 1uCi/well and incubated at 37°C for 2 hours. Following this media was removed and replaced with 200 µl of 10% (w/v) trichloroacetic acid and again incubated at 37°C for 10 minutes before being aspirated, followed by the addition of 200 µl of 1 M NaOH to each well. The plate was incubated at 37°C overnight. One hundred and fifty microlitres of the resulting solution was assessed for leucine or thymidine incorporation by scintillation counting. Results were expressed as a ratio of the thymidine/leucine incorporation normalised to the untreated control.

### Creatine kinase

The creatine kinase MB assay (Randox CK-1296) was used, all reagents were used as directed by the manufacturer and mixed with 40 µl of plasma and incubated at 25°C for 10 minutes before being transferred to a cuvette (Sarstedt, 10×4×45 mm). Absorbance at 340 nm was recorded (A1) and again 5 minutes later (A2). CK-MB activity was then calculated as described by the manufacturer.

### Creatine kinase isozyme analysis

Plasma samples containing 35 µg protein were mixed with 6× loading buffer (50% (v/v) glycerol, Tris/glycine running buffer (250 mM Tris-base, 1.9 M glycine, pH 8.6), 0.25% (w/v) xylene cyanol) and separated on 7.5% polyacrylamide (PAGE) gel, with a 3.5% stacking gel in Tris/glycine running buffer at 150 V at 4°C and transferred onto nitrocellulose membranes (Hybond-ECL, Amersham Biosciences) by semi dry blotting. After blocking in 5% (v/v) non-fat milk in TBST (10 mM Tris, 150 mM NaCl, 0.1% (v/v) Tween 20) for 2 hours, the membranes were incubated with a specific antibody to CK-MB diluted in 5% (v/v) non-fat milk in TBST (1∶200) (sc-28898, Santa Cruz Biotechnology) for 2 hours at RT. Membranes were washed three times in TBST for 5 minutes each, and incubated with anti-rabbit IgG antibody (Cell Signalling Technology) conjugated to horseradish peroxidise in 5% (v/v) non-fat milk in TBST (1∶1000) for 1 hour at RT and then washed three times with TBST for 5 minutes each. Bands were visualised by chemiluminescence (GE Healthcare), for 2 minutes without shaking. The membranes were wrapped in plastic wrap and placed in a film cassette in the dark and exposed to x-ray film (ECL hyperfilm, GE Healthcare) for 30 seconds to 2 minutes and developed. Band intensities were measured by computerized image analysis software. To assess equal protein loading, gels were stained with Coomassie Brilliant Blue R-250 (Bio-Rad) for 30 minutes while shaking and destained with 40% (v/v) methanol (Fisher Scientific) and 10% (v/v) glacial acetic acid (Fisher Scientific) solution for 1 hour, with changes to solution every 10 minutes. An image of the gel was then obtained by scanning.

### Troponin I

The mouse cardiac troponin-I (cTnI) ELISA kit (2010-1-HSP Life Diagnostics) was used to determine cTnI levels in circulating plasma. All reagents were used as supplied and directed by the manufacturer.

### RNA extraction

To each tissue sample (100 mg) 1 ml Tri Reagent was added and homogenised and incubated at RT for 5 minutes before 200 µl of 1-bromo-3-chloro-propane was added to each sample. Lysates were shaken for 20 seconds and vortexed before incubation for 3 minutes at RT. Followed by centrifugation at 13,000 rpm for 15 minutes at 4°C; this separates the lysate into two aqueous phases. The upper aqueous phase was transferred to a new tube and 600 µl of isopropanol added and incubated at RT for 10 minutes, before centrifugation at 13,000 rpm for 10 minutes at 4°C. The supernatant was removed and the resulting pellet was washed twice in 1 ml 75% (v/v) ethanol and centrifuged at 13,000 rpm for 10 minutes at 4°C. The supernatant was removed and pellets dried and suspended in 20 µl water. Extracted RNA was quantified using the NanoDrop ND-1000 UV spectrophotometer (Nanodrop technologies) and stored at −80°C.

### DNA extraction

DNA was extracted using a Qiagen kit (QIAamp ® DNA micro). All reagents were used as supplied and DNA extracted from 10 mg heart tissue.

### Transcriptional analysis

Two color microarrays with reverse labelling were carried out using a full genome mouse oligo array (70 mers), using exon centred probes (Mouse Exonic Evidence Based Oligonucleotide set (http://www.bio.davidson.edu/projects/GCAT/protocols/mouse/Oligator_MEEBO_Data_Sheet.pdf). Invitrogen), a total of 39,000 oligos were covered including spliced variants, positive, negative and doped controls. These probes were printed onto aldehyde slides (Genetix) using a Stanford type microarray spotter in house [50], [51]. Total RNA was diluted to 7 µg before the addition of 8 µg oligo dT_23_N_2_ and 10nmol random pentadecamers and incubated at 95°C for 5 minutes and 70°C for 10 minutes. Samples were then reverse transcribed with Superscript III (Invitrogen) and aadUTP was incorporated. Any remaining RNA was hydrolysed and samples neutralised by the addition of 1 M HEPES (pH 7.0). The resultant cDNA was cleaned up using Microcon YM-30 filters (Millipore), prior to conjugation with relevant Alexa fluorophores (Molecular Probes, Invitrogen). Any unbound dye was removed using the Qiagen PCR purification kit (Qiagen) according to the manufacturer's instructions. Dye incorporation was assessed using a NanoDrop ND-1000 UV (NanoDrop technologies) spectrophotometer. 2x enhanced hybridisation buffer (Genesiphere) and 4 µg Yeast tRNA (Invitrogen) was added to each sample, samples were denatured prior to loading on to the microarray and allowed to hybridize overnight at 42°C. Following hybridization slides were washed three times in 1XSSC/0.03%, room temp., 10 min/0.2XSSC room temp., 5 min./0.05%SSC room temp., 5 min. Slides were scanned using an Axon 4200A scanner with GenePix 6.0 software. For each analysis 5 biological replicates were used with two technical replicates (reverse labeling).

### Quantitative Real-Time Reverse Transcriptase PCR (qRT-PCR)

mRNA expression of selected genes were determined by qRT-PCR as described [52]using SYBR® green mastermix on an ABI PRISM® 7700 RT-PCR machine. Separate genes were run in parallel. In brief 100 ng RNA was diluted in 4.5 µl water mixed with 0.5 µl 90OD random hexamers and heated to 95°C for 5 minutes and snap cooled on ice and reversed transcribed using 8.25 µl of RT master mix (5xPCR buffer, 100 mM dATP, 100 mM dCTP, 100 mM dGTP, 100 mM dTTP, and 0.1 M DTT), 0.25 µl of RNasin (Promega), and 0.5 µl of Superscript III (Invitrogen) and incubated at 50°C for 1 hour then 70°C for 15 minutes before storage at −20°C. Primers for qRT–PCR were designed to cross exon-intron boundaries to eliminate the detection of any contaminating genomic DNA using Primer Express® software v2.0 (Applied Biosystems) and are shown in Table 1. Concentrations for each primer pair were optimised from 50 to 900 nM and the concentrations that gave the lowest C_t_ values used in the assay.

**Table 1:** Primers used for qRT-PCR.

Gene	Forward Primer	Reverse Primer
**Ndufs8**	CTGCCCTGTTGATGCCATT	TGTACAGCAACTCCTCGTGTGTCT
**Ndufa8**	GCCAACTCTGGAAGAGCTGAAA	GTGATGGGCGGCAGCTT
**Atp8a2**	TCTGCGACAACCGGATCAG	CCTAATCTGCTCATACAGGAATCG
**Atp6v1e**	GATTGACCAGGAGGCCTACCT	TTGCGATCCCCATTATAGATCTC
**Atp5h**	ACGCCAGGTTGGCTAGTCTGT	GGCCACATTGGCCCTGTAG
**Atp5g1**	CCACCAAGGCACTGCTCATT	CAGGAGGGAGGCAGACACA
**Ndufs1**	CCTAACCTCTAAGCCTTATGCCTTTA	GCATCCATTACATCAATGGACTCT
**Cox6c**	CATTGTGGCCCTGGGAGTT	TTCTGCATACGCCTTCTTTCTTG
**mt-CytB**	TTATCGCGGCCCTAGCAA	TTTAATCCTGTTGGGTTGTTTGATC
**Atp5o**	AGCTTGTAAGGCCCCCTGTT	AGAGTACAGGGCGGTTGCAT
**Ndufs2**	CGGAAATGTGACCCTCACATC	GGGCCTGCAGATAGGTCTTGT
**Tcap**	GAAGAGGGATGCTCCTTGCA	GCTGTACCACCGCCTGACA
**TBP**	GCGGTCGCGTCATTTTCT	CGGAACTCGCACAAAGCA
**Prkaa2**	CGTGTGACATTATGGCTGAAGTGTA	TACTCGAAGATGGTATGCATTCACTA
**Prkab1**	GTCATGCTGAACCACCTCTATGC	TGTACCGGTGTGTTGCACTGA
**Prkab2**	GAGCCCAATCATGTTATGCTGAA	TTGCGCTAAGGACCATCACA
**Prkaa1**	AACAGGACCAGAAAGTTATCTAGTGTGA	TGACACTTTGTTTGGCGTTTG
**Prkag3**	GACGTGCTGTATGGCAAAGTTG	TCAAGTCCAGAGGCATTTTCCT
**Prkag1**	CAGCCACCTGCCAAGCA	ACAGACCTGGACGGGTGAGTT
**Prkag2**	TTGAAGGTGTTGTGAAGTGCAAT	GATGGACCTCAGCTCTTACTATTCTGT
**mtDNA copy number**		
**ND-1**	CGGGCCCCCTTCGAC	GGCCGGCTGCGTATTCT
**ND-2**	CACGATCAACTGAAGCAGCAA	ACGATGGCCAGGAGGATAATT
**ND-3**	TGTACTCAGAAAAAGCAAATCCATATG	AATAATAGAAATGTAATTGCTACCAAGAAAAA
**ND-5**	CGGACGAACAGACGCAAATA	TAAAATGAATCCGATGTCTCCGA
**ND-6**	TTGATGGTTTGGGAGATTGGTT	TGCCGCTACCCCAATCC
**mt-CytB**	TTATCGCGGCCCTAGCAA	TAATCCTGTTGGGTTGTTTGATCC
**Cox2**	CATCCCAGGCCGACTAAATC	TTTCAGAGCATTGGCCATAGAA
**Cox3**	CAGGAT TCTTCTGAGCGTTCTATCA	AATTCC TGTTGGAGGTCAGCA
**Cox1**	GAAGAGACAGTGTTTCATGTGGTGT	TCCTGGGCCTTTCAGGAATA
**mt-ATP6**	TGTGGAAGGAAGTGGGCAA	CCACTATGAGCTGGAGCCGT

Each primer pair was optimised for concentration before use. The concentrations giving the lowest C_t_ value were used.

### Translational Profiling Using Microarrays: Sample Preparation

50 mg of heart tissue perfused with 100 µg/ml cycloheximide was homogenised in 250 µl ice cold lysis buffer (15 mM Tris-HCl pH 8, 300 mM NaCl, 15 mM MgCl_2_, 1% (v/v) Triton X-100, 100 µg/µl cycloheximide, 1 mg/ml heparin, 80 U/ml RNAsin). Samples were incubated on ice for 15 minutes followed by centrifugation at 12,000 rpm for 5 minutes at 4°C. 300 µg protein was loaded directly onto each sucrose gradient. Three animals were used with separate preparations of monosome and polysomes per time point and individual microarray analysis.

### Translational Profiling Using Microarrays: Sucrose Gradient Preparation and Centrifugation

8 ml of 65% (w/v) sucrose solution in lysis buffer (15 mM Tris-HCl pH 7.5, 300 mM NaCl, 15 mM MgCl_2_, 100 µg/µl cycloheximide, 1 mg/ml heparin, 1 mM DTT) and 8 ml lysis buffer was prepared and cooled to 4°C. A linear 10–65% sucrose gradient was prepared using a gradient station machine in Beckman ultra-clear centrifuge tubes (14×95 mm). The sample lysates (above) were loaded directly onto the top of a gradient and centrifuged at 200,000 g for 2 hours at 4°C [53]. After centrifugation gradients were fractionated by upward displacement into 1 ml aliquots. The absorbance at 254 nm was monitored throughout; samples were collected into 1 ml TRI®.

### Translational Profiling Using Microarrays: Purification of RNA and labelling

Equal amounts of RNA from appropriate fractions (i.e. polysomes or monosomes) were pooled and precipitated with 1×3 M NaOAc pH 5.0 and 3×100% (v/v) ice cold ethanol at −20°C overnight. Following centrifugation at 13,000 rpm for 15 minutes at 4°C, the resultant pellets were washed twice in 1 ml of 75% (v/v) ethanol by centrifugation at 13,000 rpm for 5 minutes at 4°C each and suspended in 10 µl of water. A direct labelling method was used with Cy3- or Cy5-dUTPs (GE Healthcare). Cy3 and Cy5 have a wavelength of 532 and 647 nm, respectively. 10 µl of the purified RNA sample (above) was incubated at 95°C for 3 minutes and snap cooled on ice for 1 minute. 1.0 µl of oligo dT_23_N_2_ (8 µg/µl) was added to each sample and incubated at 70°C for 8 minutes. 6.5 µl of direct labelling mix (dATP 0.5 mM, dGTP 0.5 mM, dCTP 0.5 mM, dTTP 0.2 mM, 1xfirst strand buffer (Invitrogen) and DTT 0.01 M) and 1 µl of Superscript III (Invitrogen), 0.5 µl of RNAsin (Promega) and 2 µl of the appropriate Cy dye were added to each sample and incubate at 50°C for 2 hours. Any remaining RNA was hydrolysed by the addition of 20.5 µl water, 1 µl of 0.5 M EDTA, 1 µl of 10% (w/v) SDS and 3 µl of 3 M NaOH to each sample and incubated at 70°C for 10 minutes and neutralised by the addition of 10 µl of 1 M Tris-HCL (pH 7.5) and 3 µl of 2 M HCL (Fisher). The resultant cDNA was purified and suspended in 20 µl water. Labelling efficiency was measured and equal amounts of labelled cDNA (Cy-3 and Cy-5) were combined. Samples were made up to a volume of 40 µl with water, 1 µl of Yeast tRNA (4 µg/µl, Invitrogen) and 40 µl of 2× enhanced cDNA hybridisation buffer (Genisphere) were added to each sample and incubated at 42°C for 30 minutes. Hybridization was conducted using a humidified chamber (Genetix Ltd, Hampshire, UK) overnight at 42°C.

### Translational Profiling Using Microarrays: Northern Blotting with Fractionated RNA

A 1% denaturing agarose gel with 18% (v/v) formaldehyde in 1xMOPS (0.2 M MOPS (pH 7.0), 80 mM NaOAc, 10 mM EDTA) was prepared by melting 80 mg agarose in 57.7 ml of water prior to the addition of 8 ml of 10xMOPS and 14.3 ml of formaldehyde and cast. Once set, the gel was submerged in 1xMOPS in an electrophoresis tank. An equal volume of each fraction (5.5 µl) was mixed 1∶1 with denaturing buffer (70% (v/v) deionised formamide, 25% (v/v) formaldehyde, 1xMOPS and 30 µg ethidium bromide) and incubated at 95°C for 15 minutes and snap cooled on ice. 2 µl RNA loading buffer (50% (v/v) deionised formamide, 1 mM EDTA, 0.25% (w/v) bromophenol blue and 0.25% (w/v) xylene cyanol) was added to each sample, prior to samples being loaded into the gel and separated overnight at 20 V. Gel images were obtained with a UVP BioImaging System (UVP, Cambridge, UK). RNA was transferred from the gel onto a positively charged nylon membrane (Genetix, New Milton, UK) by capillary transfer overnight in 3xSSC. The membrane was UV cross-linked at 120 mJ/cm^2^ for 60 seconds. Probes for β-actin were labelled with ^32^P using *E.coli* Klenow fragment, incorporation was measured by scintillation counting. Membranes were hybridised overnight at 42°C with 1×10^6^ dpm/ml probe.

### Mitochondrial complex activities

Cardiac tissue samples were prepared according to Gellerich *et al* [54]. In brief 200 mg heart tissue was homogenised in 250 µl PBS and centrifuged at 4°C for 1 minute at 6000 rpm; the resultant supernatant was stored at −80°C prior to assay. Protein concentrations of samples were determined using the Bradford assay. Measurement of all complexes except complex V is described by Barrientos (2002) [55] and assays were scaled to a 96 well plate format. Citrate synthase activity was measured according to the method of Gellerich et al. (2004) [54].

### Mitochondrial complex activities: Complex I Activity

A 1∶1 dilution of sample with 0.5% (v/v) triton X100 in PBS were prepared and incubated on ice for 1 hour. 3 µl of the resulting lysate was added to each well of a 96 flat bottomed plate (Greiner bio-one), this equated to ∼130 ug protein/well. Each sample was measured in triplicate reactions. 4 µl of 1 mM rotenone (in 100% (v/v) ethanol) was added to inhibit any complex I activity. Reaction buffer (Table 2) was added to each well. Absorbance of each 96 well plate was measured at 340 nm every 9 seconds for 10 minutes at 37°C on a SPECTRAmax PLUS 384 spectrophotometer (Molecular Devices).

**Table 2:** Reaction buffers used for mitochondrial complex activity measurements.

Reaction buffer per well			
Complex I	Complex II-III	Complex IV	Complex V
In 200 µl: 25 mM K_2_HPO_4_ titrate to pH 7.4 with KH_2_PO_4_, 12.5 ul of 50 mg/ml fatty acid free BSA, 7.5 µl of 5 mM NADH, 12.5 µl of 40 mM sodium azide, 3.75 µl of 5 mM coenzyme Q10 (in 100% (v/v) ethanol) and 13.75 µl of ddH_2_O, pre-warmed to 37°C	In 150 µl: 166 mM K_2_HPO_4_ titrate to pH 7.4 with KH_2_PO_4_, 12.5 µl of 40 mM sodium azide, 3 µl of 0.5 M succinate, 3 µl of rotenone (1 mM), 25 µl of 0.1% (w/v) BSA and 31.5 µl of 10.4 mg/ml cytochrome c, pre-warmed to 37°C	In 62.5 µl: 40 mM K_2_HPO_4_ titrate to pH 6.5 with KH_2_PO_4_, 1 M sucrose and 4 mg/ml fatty acid free BSA, 18 µl of 6.25 mM lautyl maltoside and 10 µM reduced cytochrome C in a volume of 205 µl	Per well: 20 µl of 0.5 M Tris-HCL (pH 8.0), 2 µl of 0.5 M MgCl_2_, 20 µl of 10 mM NADH, 12.5 µl of 100 mM PEP, 20 µl of 50 mg/ml fatty acid free BSA, 4 µl of 1 mM rotenone, 3 µl of 1 mM antimycin-A, 6 units of LDH and 5 units of PK and pre warmed to 37°C

Buffers used for analysis of the activity of each complex of the ETC.

### Mitochondrial complex activities: Complex II-III Activity

20 µg protein was placed in each well of a 96 well plate each sample was measured in triplicate. 18 µl of 0.5% (v/v) tween 20 in PBS was added to each well containing biological material. To one set of triplicate samples 3 µl of 1 mM antimycin-A (in 100% (v/v) ethanol) was added. In another set of triplicate samples 5 µl of 500 mM TTFA (in 100% (v/v) ethanol) was added. Reaction buffer (Table 2) was added to each well. Absorbance of each 96 well plate was measured at 550 nm every 9 seconds for 10 minutes at 37°C on a SPECTRAmax PLUS 384 spectrophotometer (Molecular Devices).

### Mitochondrial complex activities: Complex IV Activity

10 µg protein was placed in each well of a 96 well plate, each sample was measured in triplicate and each triplicate was duplicated, to the duplicate samples 25 µl of 8 mM sodium azide was added to inhibit any complex IV activity. Reaction mix (Table 2) was added to each well. Absorbance was measured at 550 nm every 9 seconds for 10 minutes at 37°C on a SPECTRAmax PLUS 384 spectrophotometer (Molecular Devices).

### Mitochondrial complex activities: Complex V Activity

Samples were homogenised in 250 mM sucrose, 2 mM EGTA, and 20 mM Tris pH 7.4 at 4°C. 15 µg protein was added into each well of a 96 flat bottomed plate. Each sample was measured in triplicate and each triplicate set was further duplicated. To the duplicate samples 3 µl of 1 mM oligomycin (in 100% (v/v) ethanol) was added to inhibit complex V activity. Reaction buffer (Table 1) was added to each well. Absorbance was measured at 340 nm every 9 seconds for 5 minutes at 37°C on a SPECTRAmax PLUS 384 spectrophotometer, this constitutes the pre-read plate. Following this 75 µl of 10 mM MgATP was added per well and again the absorbance was measured at 340 nm every 9 seconds for 10 minutes.

### Citrate Synthase Activity

100 ug protein was mixed 1∶1 with 0.5% (v/v) triton X 100 in PBS and incubated on ice for 1 hour. 3 µl of the resulting lysate was added to each well of a 96 well plate, reaction buffer (per well, containing 25 µl of 0.4 mg/ml DTNB (dissolved in 1 M Tris-HCl pH 8.1), 7.5 µl of 10 mg/ml acetyl CoA (dissolved in acidified water pH 5.0) and 165 µl water) was added to each well. Absorbance was measured at 412 nm every 9 seconds for 3 minutes on a SPECTRAmax PLUS 384 spectrophotometer, this constitutes the pre read plate. Following this 12.5 µl of 0.5 mM oxaloacetic acid was added per well and the plate was again read at 412 nm every 9 seconds for 5 minutes. Prior to calculation of activity the pre-read plate Vmax was subtracted from the post oxaloacetic acid plate.

### Mitochondrial complex activities: Calculation of activity

Complex and citrate synthase activity was calculated for each sample when the change in absorbance against time was linear. This change was taken as the maximum rate of reaction (Vmax) where the regression value is equal to 1. The absorbance of the inhibited samples was subtracted from the corresponding non-inhibited samples to obtain the Vmax of complex activity in each sample. Data was normalized to the amount of protein in each sample and expressed as ΔAbs/min(Vmax)/ug protein. The inverse Vmax/ug protein was plotted to avoid confusion over the change in activity.

### Measurement of ATP/ADP/AMP Activity by HPLC

50 mg of heart tissue was homogenised in 1 ml of 10% (v/v) perchloric acid. Samples were centrifuged at 10,000 rpm for 15 minutes at 4°C. Internal standards were added and the resulting supernatant neutralised with 1 M KOH and subjected to HPLC [56]. HPLC was performed on a BDS-Hypersil 250×4.6 mm C_18_ 5 µm column using a Transgenomic HPLC Analyser. Buffer A was 83.3 mM triethylammonium phosphate (TEAP) pH 6.0 and Buffer B was HPLC grade methanol. The flow rate was set at 1 ml/min. The injection volume was 10 µL. ATP/ADP/AMP was monitored at 206 nm and the amount of ATP/ADP/AMP in each sample determined by calibration of the system with standards of known concentration and the data analysed with reference to the standards and expressed as µM/g tissue.

### Caspase 3

Caspase 3 activity was measured using a colorimetric assay according to the manufacturer's instructions (Clontech). For this assay 10 mg of heart tissue was homogenized in the kit lysis buffer**.**

### mtDNA copy number

Genomic DNA (100 ng) was isolated using QIAamp® DNA micro kit (Qiagen) and was subjected to amplication with SYBR® green mastermix on an ABI PRISM® 7700 RT-PCR machine as described above. Primers were designed using Primer Design software. Data was normalized to that of a nuclear encoded gene. Relative quantification was performed with the comparative cycle threshold method (Applied Biosystems, User Bulletin No. 2, 1997).

### Methemoglobin assay

Methemoglobin levels were determined in whole blood according to the method of Anders and Chung 1984 [57]. In duplicate 25 µl of blood was mixed with 2 mls of water and incubated at RT. Following incubation 25 µl of 30 g/L hydrogen peroxide was added to one of the samples (A1) and 10 µl of 50 g/L sodium nitrite to the other (A2). 8 ml PBS (pH 6.6) was added to all samples and 1 ml of each sample placed into a cuvette (Sarstedt, 10×4×45 mm). 5 ml of the control sample was further diluted with an additional 5 ml PBS (pH 6.6) and 1 ml placed in a cuvette (Sarstedt, 10×4×45 mm); this sample was used as a blank. The absorbance at 405 nm was recorded. Results were calculated as follows: methemoglobin % = (A1/A2)×100.

### Imaging

HL-1 cells were simultaneously stained with 0. 8 µM Hoechst and 100 nM Mitrotracker deep red (Invitrogen) diluted in claycomb media for 1 hour at 37°C. Following incubation cells were washed twice with PBS, before being fixed and permeabilized with 3.7% formaldehyde and 0.2% triton x100 for 20 minutes, again at 37°C. Fixative was removed and again the cells were washed twice with PBS prior to blocking in 1.1% BSA and 0.2% triton x100 for 30 minutes in the dark. Blocking solution was removed and replaced with 1∶100 dilution of sarcomeric α-actinin (ab9465, abcam) in blocking buffer. Cells were incubated at 37°C for 2 hours. Following incubation the primary antibody was removed and cells were washed in PBS containing 0.2% triton x100. Following washing cells were again incubated in blocking buffer for 30 minutes, before the addition of the secondary antibody (goat anti-mouse Alexa fluor 488, cat# A11001, Invitrogen) diluted 1∶500 in blocking buffer for 1 hour at 37°C. Subsequently cells were washed twice in PBS before image capture using an Imagexpress system (Molecular Devices). Automated image analysis was conducted using Metaexpress (Molecular Devices). Both the primary and secondary antibody were incubated with cells alone to check for cross reactivity.

### Statistical and bioinformatic analysis

Microarray data was LOWESS normalized using ArrayTrack (http://www.fda.gov/ScienceResearch/BioinformaticsTools/Arraytrack/default.htm) and pathway analysed using the Gene Set Analysis Toolkit, KEGG pathway feature (http://bioinfo.vanderbilt.edu/webgestalt). The significance of each KEGG pathway was assessed using a modified Fisher exact test, this generates an enrichment probability score (P value) [58]. All other data is expressed as the mean ± SD of three to five independent experiments with two technical replicates in each group. The data were analyzed using one-way ANOVA, differences between individual means were compared using a Dunnett's post-hoc test. A probability of P≤0.05 was used as the criterion for significance. The full dataset has been submitted to GEO accession number GEO18459.

## Supporting Information

Figure S1 Principal components analysis of the transcriptional data for DOX and DMNQ.
(4.33 MB EPS)

Figure S2 Activation of transcription factor NRF-1 with DOX and DMNQ.
(4.75 MB EPS)

Figure S3 Hierarchical clustering of genes regulated by NRF-1 with time after DOX (left) or DMNQ (right) treatment *in vivo*.
(8.89 MB EPS)
