# Correction: Doxorubicin *In Vivo* Rapidly Alters Expression and Translation of Myocardial Electron Transport Chain Genes, Leads to ATP Loss and Caspase 3 Activation

**DOI:** 10.1371/journal.pone.0102278

**Published:** 2014-07-02

**Authors:** The PLOS ONE Staff

Figure 1F in the article shows the changes in plasma creatine kinase isozymes following the administration of 2,3-dimethoxy-1,4-naphthoquinone to mice as a single dose (25mg/kg ip) determined by native protein gels followed by detection using Western analysis and an antibody against creatine kinase. The lower panel in the figure displays Coomassie blue staining to determine equal protein loading, this panel is incorrect in the published Figure 1F, it displays a duplication of the first two lanes. The published Figure 1F should also have indicated that the image was generated using lanes from two separate blots.

**Figure 1:**
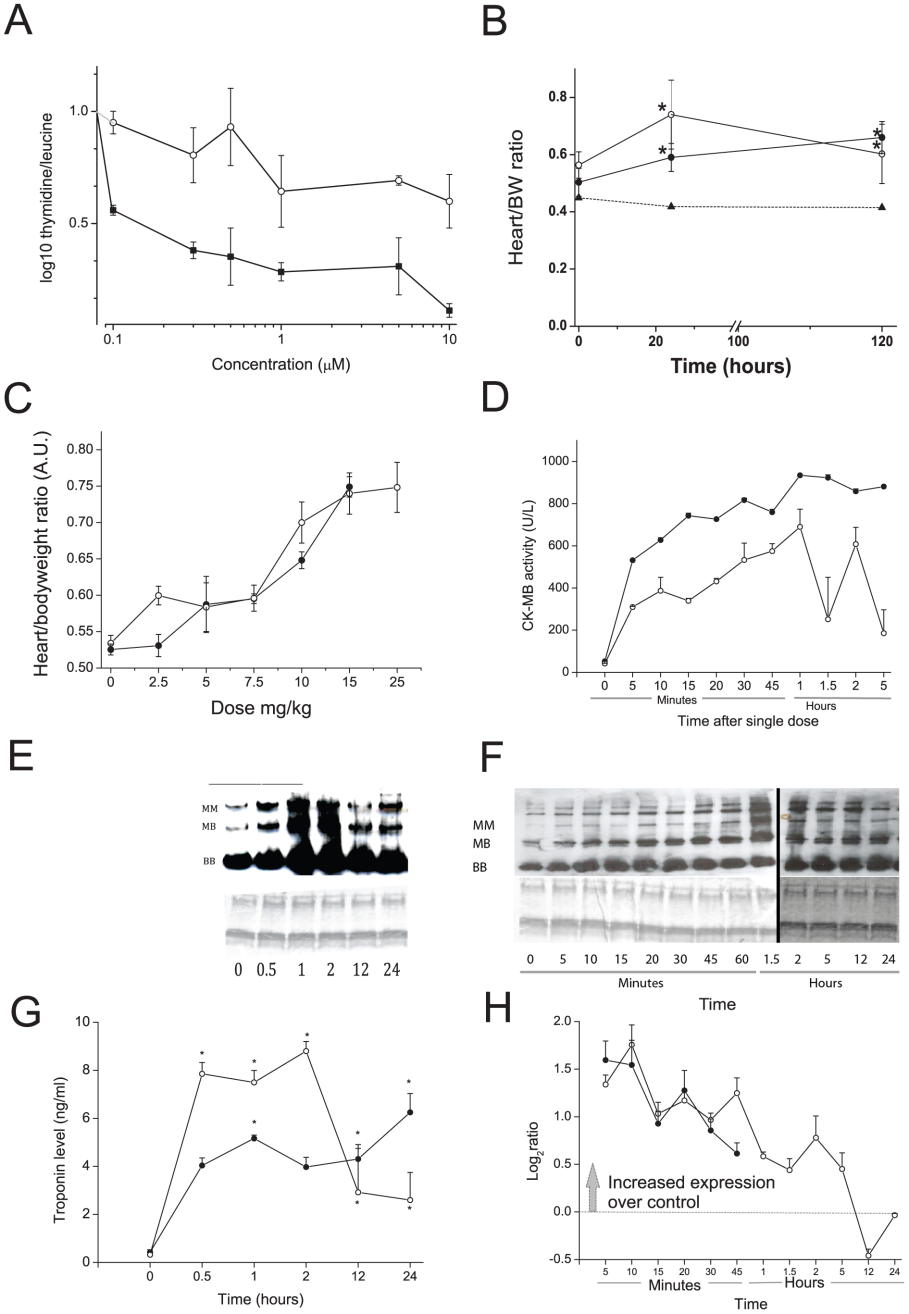
Determination of Cardiac Damage. A) Effect of DOX (filled circles) and DMNQ (open circles) on incorporation of 3H-thymidine/3H-leucine (open circles) in the mouse cardiac cell line HL-1. Incorporation was measured after 24 hr exposure to the indicated concentration. DOX significantly different from DMNQ p  =  0.003 two way ANOVA. B) Heart: body weight ratio change with time after a single acute dose of DOX (15 mg/kg, filled circle) or DMNQ (25 mg/kg, open circle), or vehicle (triangle). C) Heart:body weight ratio following a dose response 120 hrs post dosing (DOX filled circles and DMNQ open circles). D) CK-MB activity determined from plasma following DOX (filled circles) or DMNQ (open circles). E and F) Native protein gels DOX (E) and DMNQ (F)probed for CK. Coomassie blue stain indicated equal protein loading. G) Troponin I activity determined from plasma following DOX (filled circles) or DMNQ (open circles) using an ELISA assay kit. H) TCap mRNA levels from the microarray data over time after acute DOX (filled circles) or DMNQ (open circles). In all graphs mean and SD are plotted. For DOX in D the SDs are very small and hidden behind the points. B,D and G: Statistical analyses performed using a one way ANOVA to time 0 with Dunnetts post-hoc t-test. *  =  p<0.05, except D where all points were significant.

We are providing a revised Figure 1F with the corrected lower panel, and where the non-adjacent lanes are denoted by vertical lines.

## References

[1] Pointon AV, Walker TM, Phillips KM, Luo J, Riley J, et al (2010) Doxorubicin In Vivo Rapidly Alters Expression and Translation of Myocardial Electron Transport Chain Genes, Leads to ATP Loss and Caspase 3 Activation. PLoS ONE 5(9): e12733 doi:10.1371/journal.pone.0012733 20856801 10.1371/journal.pone.0012733 PMC2939875

